# Dissipation in Lagrangian Formalism

**DOI:** 10.3390/e22090930

**Published:** 2020-08-25

**Authors:** András Szegleti, Ferenc Márkus

**Affiliations:** Department of Physics, Budapest University of Technology and Economics, 1111 Budapest, Hungary; markus@phy.bme.hu

**Keywords:** harmonic oscillator, calculus of variations, Lagrangian mechanics, Lagrangian framework, Hamiltonian mechanics, dissipation

## Abstract

In this paper, we present a method by which it is possible to describe a dissipative system (that is modeled by a linear differential equation) in Lagrangian formalism, without the trouble of finding the proper way to model the environment. The concept of the presented method is to create a function that generates the measurable physical quantity, similarly to electrodynamics, where the scalar potential and vector potential generate the electric and magnetic fields. The method is examined in the classical case; the question of quantization is unanswered.

## 1. Introduction

Newtonian mechanics can provide a general description of a physical system, as there are no limitations on the force terms that can contribute to the equations of motion. This freedom is actually a drawback of formalism, as correct equations of motion can be generated using ad hoc forces, which limits the possibility of gaining predictions from the theory.

Lagrangian formalism is built on a more general principle, Hamilton’s principle (or least action principle), which states that there is a function $L(q_{i},{\dot{q}}_{i},t)$, that describes the physical system, and the action functional

(1) $$S=\int_{t_{1}}^{t_{2}}dtL\left(q_{i},{\dot{q}}_{i},t\right)$$

is extremal in the case of physical trajectories. The equations of motion (Euler–Lagrange equations) for the system can be calculated from variational principle (functional derivative). This approach strongly limits the form of equations that one can derive using this formalism. At this point, some kind of generalization is necessary if one wishes to obtain a differential equation that cannot be derived from a Lagrangian. One way is to generalize the functional derivative to a fractional order [1,2,3,4,5,6]. Another way is to increase the degrees of freedom, which is the basis of many different methods.

Different approaches have tried to grab the essential points to achive a formulation of dissipative processes in Lagrangian formalism. Nowadays, the study is again at the center of researcher interest, but it is important to note that it has a long history. First, it was Rayleigh [7] who investigated the possibility of describing dissipation (due to viscosity) within Lagrangian formalism, by adding a so-called dissipation function to the Euler–Lagrange equations. He concludes that the extra term cannot be included in the Lagrangian, meaning that it cannot be obtained from variational principle. Building on this idea, the principle of the least dissipation of energy is formulated and used for irreversible processes and non-equilibrium thermodynamics [8,9,10,11]. In later works [12] however, Rayleigh’s concept is incorrectly considered as a variational method. As mentioned before, there is a strict limitation of the form of Euler–Lagrange equations, indicating that this approach cannot be successful.

Dissipation, being a statistical phenomenon, could only be described by a Lagrangian containing all degrees of freedom (both for the system and its environment). This approach is successful for both classical and quantum systems. Using this system-plus-reservoir approach, how the environment should be modeled is always an important question. For example, using a harmonic bath model [13], one assumes that the reservoir can be represented as a set of uncoupled harmonic oscillators. By using a different model for the environment, a set of two-state systems [14,15], the resulting dynamics of the system might be different.

Despite being conceptually incorrect in the sense that the environment is not modeled properly, it might be satisfying for our needs to neglect the environment and use a mathematical trick that ensures the correct form of equations for the system of interest. One such way of doing so is using an explicitly time-dependent Lagrangian, as the resulting equations of motion will show a dissipation of energy. For the damped linear harmonic oscillator, there exists a widely known Lagrangian proposed by Bateman [16], from which the correct equation of motion can be obtained. Quantization of the system, first performed by Caldirola [17] and Kanai [18] independently, turned out to be problematic, as the quantization of an explicitly time-dependent Hamiltonian results in conflicts with the uncertainty principle [19,20]. Another approach to construct a Lagrangian that can produce Euler–Lagrange equations that describe dissipation, also introduced by Bateman [16], is doubling degrees of freedom, by defining a mirror system which the system of interest is coupled to. In a sense, this mirror system is an oversimplified model of the environment. The energy of the system decreases, while that of the mirror system increases in time, and the total energy is constant.

This may give the idea that there might be a workaround by which it is possible to describe dissipation phenomena (more generally than dissipation of energy), without the trouble of finding a correct model for the environment. One way of doing this (at least in principle), is to define a potential (by doubling the degrees of freedom) that generates physical quantity. It is important to point out that the question of quantization is unanswered.

## 2. Formulating the Method

In this method, the potential is a function which does not necessarily carry any physical meaning, but contains all the physical information, and it thus generates an observable physical quantity. It can be a purely mathematical tool to build Lagrangian formalism that will generate the desired equation of motion for the observable in the end. It is possible to define a potential for any quantity described by any linear differential equation [21,22,23,24]. The idea is similar to how one deals with observables and potentials in electrodynamics, although there are major differences, such as that there are no undesirable solutions in electrodynamics. The observables (electric field and magnetic flux density) cannot be handled in Lagrangian formalism, but potentials (scalar potential and vector potential) can be defined, with corresponding differential equations of a higher order.

### 2.1. Creating a Lagrangian Using Potentials

A general linear Euler–Lagrange equation can be written in the form

(2) $$\begin{array}{c} \tilde{D}\left\{\frac{\partial L}{\partial Du}\right\}=0 \end{array}$$

where D is a formal linear differential operator and $\tilde{D}$ is its formal adjoint defined by

(3) $$\begin{array}{c} \int_{\Omega }d\tau \;v\cdot Du-\int_{\Omega }d\tau \;u\cdot \tilde{D}v=\int_{\partial \Omega }d\nu \;B\left\{u,v\right\}, \end{array}$$

where $B\left\{u,v\right\}$ is called the bilinear concomitant. This definition provides the possibility of calculating $\tilde{D}u$ through repeated integration by parts. Let’s look at the example of a general differential operator of order *n* acting on a function with a single variable

(4) $$\begin{array}{c} Du=p_{n}\frac{d^{n}u}{dt^{n}}+p_{n-1}\frac{d^{n-1}u}{dt^{n-1}}+\ldots +p_{1}\frac{du}{dt}+p_{0}u, \end{array}$$

for which the adjoint operator acting on the function is

(5) $$\begin{array}{c} \tilde{D}u={\left(-1\right)}^{n}\frac{d^{n}}{dt^{n}}\left(p_{n}u\right)+{\left(-1\right)}^{n-1}\frac{d^{n-1}}{dt^{n-1}}\left(p_{n-1}u\right)+\ldots . \end{array}$$

We can say that D is self-adjoint if $Du\equiv \tilde{D}u$.

Suppose that a measurable physical quantity u(t) is described by the following inhomogeneous equation

(6) $$\begin{array}{c} Du(t)=c(t), \end{array}$$

where c(t) is arbitrary function. If D is not self-adjoint, it cannot be calculated from variational principle. One can define the potential ϕ(t) through the definition equation

(7) $$\begin{array}{c} u\left(t\right)=\tilde{D}\phi \left(t\right). \end{array}$$

Substitute Equation (7) in Equation (6) and

(8) $$\begin{array}{c} D\tilde{D}\phi \left(t\right)=c\left(t\right). \end{array}$$

is received. By using Equation (3), it is easy to see that the differential operator $D^{\prime }:=D\tilde{D}$ is self-adjoint, hence the equation of motion for the potential ϕ can be calculated from variational principle, so a Lagrangian exists, from which the equation of motion (8) can be calculated. This Lagrangian can be written in the following form

(9) $$\begin{array}{c} L=\frac{1}{2}(\tilde{D}\phi \left(t\right))\cdot (\tilde{D}\phi \left(t\right))-\phi \left(t\right)\cdot c\left(t\right). \end{array}$$

By using Equation (2), the Euler–Lagrange equation can be calculated, resulting in Equation (8).

### 2.2. On the Solutions

The potential ϕ(t) contains all physical information, and some excess, non-physical information can be encoded in it as well. Consider the linear operator D and its adjoint $\tilde{D}$, and suppose that $D,\tilde{D}\in \mathrm{Lin}(V)$. As it is possible to obtain the original differential Equation (6) from Equation (8), it is safe to say that the kernel of $\tilde{D}$ contains only non-physical information. By writing the solution ϕ(t) in such a way that ϕ(t)=φ(t)+λ(t), where D˜φ(t)∈Im(D˜) and $\lambda \left(t\right)\in \mathrm{Ker}\left(\tilde{D}\right)$, it is easy to see that the λ(t) term can be omitted

(10) $$\begin{array}{cc} Du(t) & =D\tilde{D}\phi \left(t\right)=D\tilde{D}(\varphi \left(t\right)+\lambda \left(t\right))\\ & =D\left(\tilde{D}\varphi \left(t\right)+\tilde{D}\lambda \left(t\right)\right)=D\tilde{D}\varphi \left(t\right). \end{array}$$

This can be interpreted as a kind of gauge freedom, because by omitting the $\lambda \left(t\right)\in \mathrm{Ker}\left(\tilde{D}\right)$ part of the potential, the measurable physical quantity will stay invariant, so one can define the gauge transformation as

(11) $$\phi \left(x\right)\to \phi \left(x\right)+\Lambda \left(x\right)\;\mathrm{where}\;\Lambda \left(x\right)\in \mathrm{Ker}\left(\tilde{D}\right).$$

The solution of the adjoint equation $\tilde{D}\lambda \left(t\right)=0$ is related to the time-reversed process. Consider a homogeneous ordinary differential equation with constant coefficients, for which the differential operator is

(12) $$D=\sum_{n=0}^{N}p_{n}\frac{d^{n}}{dt^{n}},$$

The adjoint equation reads

(13) $$\tilde{D}\zeta \left(t\right)=\sum_{n=0}^{N}{\left(-1\right)}^{n}p_{n}\frac{d^{n}\zeta \left(t\right)}{dt^{n}}=0.$$

It can be seen, that every odd-order derivative changes its sign, and the even-order terms are invariant. By changing the sign of the variable *t* (t→−t), the adjoint equation can be rewritten

(14) $$\sum_{n=0}^{N}{\left(-1\right)}^{n}p_{n}\frac{d^{n}\zeta \left(-t\right)}{dt^{n}}\frac{1}{{\left(-1\right)}^{n}}=\sum_{n=0}^{N}p_{n}\frac{d^{n}\zeta \left(-t\right)}{dt^{n}}=D\zeta \left(-t\right)=0.$$

In such a simple case, it can be clearly seen, that if ζ(t) is a solution of $\tilde{D}\zeta \left(t\right)=0$, then its time reversed is a solution of Dζ(−t)=0. As a consequence, $\lambda \left(t\right)\in \mathrm{Ker}\tilde{D}$ is related to the time reversed of v(t)∈KerD. Dissipative processes in nature tend to an equilibrium state, so the time reversed of these solutions is divergent. To obtain a stable solution, the divergent term ($\lambda \left(t\right)\in \mathrm{Ker}\tilde{D}$) should be omitted.

### 2.3. On the Initial Conditions

In theory, it is easy to omit the solutions from $\mathrm{Ker}\tilde{D}$, and for an analytical solution, one can easily perform the correct gauge transformation. Unfortunately, it does not seem possible if we wish to solve the differential equation numerically. A good idea would be to choose the initial and boundary value conditions carefully, so that the non-physical part λ(t) vanishes. The aim is to find the relation between the initial conditions for the potential and the initial conditions for the measurable.

For the sake of simplicity, let’s deal with only one variable. Firstly, write the general solution for the inhomogeneous equation Equation (8) in the form

(15) $$\begin{array}{c} \phi \left(t\right)=\sum_{k=1}^{N}[a_{k}\varphi_{k}\left(t\right)+b_{k}\lambda_{k}\left(t\right)]+\xi \left(t\right), \end{array}$$

where $\tilde{D}\varphi_{k}\left(t\right)$ form the basis for the subspace Ker(D) and $\lambda_{k}\left(t\right)$ form the basis for the subspace $\mathrm{Ker}(\tilde{D})$ and ξ(t) is a particular solution of the inhomogeneous equation (so the solution ϕ(t)=φ(t)+λ(t) is expanded on a basis). To solve a differential equation of order 2N, we need 2N initial conditions. As the number of initial conditions and the number of coefficients ($a_{k}$ and $b_{k}$) are the same, a unique solution exists. Physics provides only half of it, so we have to come up with the other half in a way that ensures the vanishing of all $b_{k}$ coefficients in Equation (15). The general form of the measurable is

(16) $$\begin{array}{c} u\left(t\right)=\sum_{k=1}^{N}a_{k}v_{k}\left(t\right)+w\left(t\right), \end{array}$$

where $v_{k}\left(t\right)=\tilde{D}\varphi_{k}\left(t\right)$, which is a basis in Ker(D).

It is possible to create the initial conditions for the measurable from the initial conditions for the potential. Let the initial conditions for the potential be

(17) $$\begin{array}{c} \phi_{0,n}=\frac{d^{n-1}\phi }{dt^{n-1}}{|}_{t=0}\;\mathrm{where}\;n=\left\{1,\;2,\;\ldots 2N\right\}, \end{array}$$

and let the initial conditions for the measurable be

(18) $$\begin{array}{c} u_{0,n}=\frac{d^{n-1}u}{dt^{n-1}}{|}_{t=0}\;\mathrm{where}\;n=\left\{1,\;2,\;\ldots N\right\}. \end{array}$$

The initial conditions for the measurable can be obtained by a linear combination of the initial conditions for the potential. It can be proven by a straightforward calculation:

(19) u0,n=dn−1dtn−1D˜ϕ|t=0=dn−1dtn−1∑i=0N(−1)ididti(piϕ)t=0=dn−1dtn−1∑i=0N∑l=0i(−1)iildldtlpi·di−ldti−lϕt=0=∑i=0N∑l=0i∑m=0n−1Tn,i,l,mϕ0,n+i−l−m,

where

(20) Tn,i,l,m=(−1)iiln−1mdl+mdtl+mpi|t=0.

Unsurprisingly, this relation cannot be inverted, but it provides a limitation on the configuration of the potential initial conditions.

One possible (but not effective) way to find correct initial conditions for the potential in the numerical simulation is to try random configurations that reproduce the physical initial conditions (this can be checked using Equation (19)). The closer the system starts in the phase space to the configuration that ensures the vanishing of the non-physical part, the slower the divergent part of the solution will start to dominate. Other than trying, it seems improbable, that there is a method to create the desired initial conditions.

### 2.4. Theoretical Background of Higher-Order Lagrangian and Hamiltonian Formalism

For physical systems, the described Lagrangian has the form $L(t,q_{i},{\dot{q}}_{i})$, so it contains at maximum first-order time derivatives of the generalized coordinates. The instability theorem of Ostrogradski [25] implies that, for a non-degenerate Lagrangian describing a physical system, dependence on a higher than first-order time derivative leads to a linear instability in the respective Hamiltonian. Of course, for a mathematical construction, this instability might not be problematic, and causes no contradiction to the observed behavior of physical systems. As for the case of abstract potential, the equation of motion for the potential can be obtained from a Lagrangian depending on higher-order time derivatives of the generalized coordinates. Firstly, this means that the potential is not a measurable physical quantity (this will ensure that there is no contradiction with Ostrogradski’s theorem); secondly, a generalization of variational principle [26] is necessary for the method to use. In this subsection, the results from [26,27] are presented.

For a Lagrangian of the form $L(t,q_{i},{\dot{q}}_{i},{\ddot{q}}_{i},\ldots )$, the equations of motion can be derived using that the first functional derivative of the action which should be vanishing, leading to

(21) $$0=\sum_{n=0}^{N}{\left(-1\right)}^{n}\frac{d^{n}}{dt^{n}}\frac{\partial L}{\partial \left(\frac{d^{n}}{dt^{n}}q_{i}\right).}$$

Building Hamiltonian formalism is possible by correctly choosing canonical coordinate and momentum pairs

(22a) $$\begin{array}{cc} q_{i,n} & :=\frac{d^{n-1}}{dt^{n-1}}q_{i} \end{array}$$

(22b) $$\begin{array}{cc} p_{i,n} & :=\sum_{k=0}^{N-n}{\left(-1\right)}^{k}\frac{d^{k}}{dt^{k}}\frac{\partial L}{\partial (\frac{d}{dt}q_{i,n+k})}, \end{array}$$

where n=1,…N. In that case, the Hamiltonian can be calculated from the Lagrangian

(23) $$H=\sum_{i}\left(p_{i,1}\frac{dq_{i,1}}{dt}+p_{i,2}\frac{dq_{i,2}}{dt}+\ldots +p_{i,N}\frac{dq_{i,N}}{dt}\right)-L$$

and the canonical equations can be obtained in the usual form

(24a) $$\begin{array}{cc} \frac{dq_{i,n}}{dt} & =\frac{\partial H}{\partial p_{i,n}} \end{array}$$

(24b) $$\begin{array}{cc} \frac{dp_{i,n}}{dt} & =-\frac{\partial H}{\partial q_{i,n}}. \end{array}$$

In the Hamiltonian, there are no higher-order derivatives; the canonical equations are first order and the dimension of the phase space is 2·M·N, where *M* is the number of general coordinates and *N* is the order of the highest order derivative present in the Lagrangian. Notice that the Lagrangian depends on M·(N+1) variables, so the Hamiltonian depends on M·(N−1) extra variables not present in the Lagrangian. These terms are present at the first power in the Hamiltonian, leading to a linear instability addressed in Ostrogradski’s theorem.

## 3. Application for the Damped Linear Harmonic Oscillator

The damped harmonic oscillator is a really good toy model to test different methods on. The undamped harmonic oscillator is a well-known system, both classically and quantum mechanically, so it provides a good starting point for introducing the damping. Furthermore, the described equation of motion can be a result of a Fourier transform in the space variable on a partial differential equation (e.g., telegraph equation). The equation of motion for the damped harmonic oscillator is

(25) $$m\ddot{x}+2m\lambda \dot{x}+m\omega^{2}x=0,$$

where *m* is the mass, λ is the damping coefficient and ω is the angular frequency.

To define a potential *q* for the measurable quantity *x*, the adjoint equation must be calculated first. As the coefficients are constant, this can be easily done, and the definition equation can be obtained

(26) $$x=\ddot{q}-2\lambda \dot{q}+\omega^{2}q.$$

By following the method, described in Section 2.1, the following Lagrangian is received:

(27) $$L=\frac{1}{2}{\left(\ddot{q}-2\lambda \dot{q}+\omega^{2}q\right)}^{2}.$$

The method guarantees that the Euler–Lagrange equation will be

(28) $$(\frac{d^{2}}{dt^{2}}+2\lambda \frac{d}{dt}+\omega^{2})(\frac{d^{2}}{dt^{2}}-2\lambda \frac{d}{dt}+\omega^{2})q=0.$$

### 3.1. Underdamped and Overdamped Cases

As the coefficients are constants in the differential operator, it will commute with its adjoint, which means that the solution for q(t) can be easily calculated

(29) $$q\left(t\right)=a_{1}e^{-(\lambda +\gamma )t}+a_{2}e^{-(\lambda -\gamma )t}+b_{1}e^{(\lambda +\gamma )t}+b_{2}e^{(\lambda -\gamma )t},$$

where $\gamma =\sqrt{\lambda^{2}-\omega^{2}}$. The terms proportional to $e^{\lambda t}$ are solutions of the adjoint operator, hence they are non-physical solutions, and therefore they will not contribute to the measurable x(t). The effect of the adjoint operator on the other two terms is just a multiplication by a constant value, so they are two independent solutions of the original differential operator.

Physics provides the initial conditions for the measurable variables

(30) $$\begin{array}{c} x\left(t=0\right)=x_{0}, \end{array}$$

(31) $$\begin{array}{c} \dot{x}\left(t=0\right)=v_{0}. \end{array}$$

By choosing the initial conditions for the potential

(32) $$\begin{array}{cc} q(0) & =\frac{2\lambda x_{0}+v_{0}}{4\lambda (\lambda^{2}-\gamma^{2})}, \end{array}$$

(33) $$\begin{array}{cc} \dot{q}\left(0\right) & =-\frac{x_{0}}{4\lambda }, \end{array}$$

(34) $$\begin{array}{cc} \ddot{q}\left(0\right) & =-\frac{v_{0}}{4\lambda }, \end{array}$$

(35) $$\begin{array}{cc} \dddot{q}\left(0\right) & =\frac{\left(\lambda^{2}-\gamma^{2}\right)x_{0}+2\lambda v_{0}}{4\lambda }, \end{array}$$

the non-physical solutions (the exponentially increasing terms in Equation (29)) will vanish, so the coefficients will be

(36) $$\begin{array}{cc} a_{1} & =\frac{\left(\gamma -\lambda \right)x_{0}-v_{0}}{8\gamma \lambda (\lambda +\gamma )}, \end{array}$$

(37) $$\begin{array}{cc} a_{2} & =\frac{\left(\gamma +\lambda \right)x_{0}+v_{0}}{8\gamma \lambda (\lambda -\gamma )}, \end{array}$$

(38) $$\begin{array}{cc} b_{1} & =0, \end{array}$$

(39) $$\begin{array}{cc} b_{2} & =0. \end{array}$$

### 3.2. Critical Damping and Undamped Case

There are 2 interesting cases, when the characteristic equation of the differential equation Equation (28) has repeated roots, λ=ω and λ=0. For λ=ω, the equation of motion is

(40) $$\frac{d^{4}q}{dt^{4}}-2\omega^{2}\frac{d^{2}q}{dt^{2}}+\omega^{4}q=0,$$

for which the general solution and the measurable variable are

(41) $$q=c_{1}e^{-\omega t}+c_{2}te^{-\omega t}+c_{3}e^{\omega t}+c_{4}te^{\omega t},$$

(42) $$x=e^{-\omega t}\left(4c_{1}\omega^{2}-4c_{2}\omega +4c_{2}\omega^{2}t\right).$$

Here, the terms proportional to $e^{\omega t}$ will not contribute to the measurable variable, so they will not carry any physical information. This is similar to the previous cases where the exponentially increasing terms were solutions of the adjoint operator. This means that only the decreasing terms are enough to construct a potential carrying all physical information. We can choose the initial conditions for the potential in the following way

(43) $$\begin{array}{cc} q(0) & =\frac{2\omega x_{0}+v_{0}}{4\omega^{3}}, \end{array}$$

(44) $$\begin{array}{cc} \dot{q}\left(0\right) & =-\frac{x_{0}}{4\omega }, \end{array}$$

(45) $$\begin{array}{cc} \ddot{q}\left(0\right) & =-\frac{v_{0}}{4\omega }, \end{array}$$

(46) $$\begin{array}{cc} \dddot{q}\left(0\right) & =\frac{\omega x_{0}+2v_{0}}{4}, \end{array}$$

it will ensure the vanishing of the non-physical solutions and will result in the following values of the coefficients $c_{i}$

(47) $$\begin{array}{cc} c_{1} & =\frac{2\omega x_{0}+v_{0}}{4\omega^{3}}, \end{array}$$

(48) $$\begin{array}{cc} c_{2} & =\frac{\omega x_{0}+v_{0}}{4\omega^{2}}, \end{array}$$

(49) $$\begin{array}{cc} c_{3} & =0, \end{array}$$

(50) $$\begin{array}{cc} c_{4} & =0. \end{array}$$

Interestingly, something unexpected occurs if the λ=0 case is investigated. The equation of motion for this special case is

(51) $$\frac{d^{4}q}{dt^{4}}+2\omega^{2}\frac{d^{2}q}{dt^{2}}+\omega^{4}q=0,$$

for which the general solution and the measurable variable are

(52) $$q=c_{1}e^{-i\omega t}+c_{2}te^{-i\omega t}+c_{3}e^{i\omega t}+c_{4}te^{i\omega t},$$

(53) $$x=-c_{2}2i\omega e^{-i\omega t}+c_{4}2i\omega e^{i\omega t}.$$

As can be seen, only the polynomially increasing terms carry physical information. This might lead to the assumption that if the information is encoded in increasing terms of the general solution, the system is not dissipative. However, the validity of this assumption can be brought into question.

In this case, it is also possible to choose the initial conditions, so the non-physical terms will vanish. The correct choice is

(54) $$\begin{array}{cc} q(0) & =0, \end{array}$$

(55) $$\begin{array}{cc} \dot{q}\left(0\right) & =-\frac{v_{0}}{2\omega^{2}}, \end{array}$$

(56) $$\begin{array}{cc} \ddot{q}\left(0\right) & =x_{0}, \end{array}$$

(57) $$\begin{array}{cc} \dddot{q}\left(0\right) & =\frac{3v_{0}}{2}, \end{array}$$

by which the coefficients $c_{i}$ are

(58) $$\begin{array}{cc} c_{1} & =0, \end{array}$$

(59) $$\begin{array}{cc} c_{2} & =\frac{-v_{0}+i\omega x_{0}}{4\omega^{2}}, \end{array}$$

(60) $$\begin{array}{cc} c_{3} & =0, \end{array}$$

(61) $$\begin{array}{cc} c_{4} & =\frac{-v_{0}-i\omega x_{0}}{4\omega^{2}}. \end{array}$$

Thus, we can conclude that a contradiction free Lagrangian formulation of the damped harmonic oscillator is elaborated.

## 4. Discussion

By creating a potential, linear differential equations describing a dissipative system can be calculated from a Lagrangian. Using the described method, the potential can be easily constructed to an equation that expresses the dissipative behavior of a physical quantity. The problem of properly modeling the environment vanishes, and instead, the adjoint equation (which defines the connection between the potential and the measurable) must be solved. Although in theory, it is possible to omit non-physical solutions which result in instabilities, technically there is no way to do that if the equation cannot be solved analytically. Moreover, if initial conditions that provide a zero non-physical part in the solution are found during a numerical simulation, numerical errors can result in an unstable solution. One proper way to stabilize such a simulation is to find a relation that can be checked throughout the solving procedure and that restricts the solution to the physical part only. Perhaps, by using Ostrograski’s instability theorem in a clever way, it could be possible to find a way to eliminate the non-physical solution from the Hamiltonian more easily than from the Lagrangian.

The benefit of this method not only lies in the fact that it provides a way of receiving an equation from a Lagrangian, but it provides the powerful tools of the Lagrangian framework, such as symmetries, possibility of quantization, coupling different systems, etc. Of course, it is an open question of how useful these tools are on the level of potentials, and how the physical information is obtained. One highly interesting idea is quantization, if it is even possible through this method. Another exciting utilization is coupling fields [28].
